# First insights into the prevalence, genetic characteristics, and pathogenicity of *Bacillus cereus* from generations worldwide

**DOI:** 10.1128/msphere.00702-24

**Published:** 2024-10-23

**Authors:** Cuihong Tong, Danyu Xiao, Qi Li, Jing Gou, Shuang Wang, Zhenling Zeng, Wenguang Xiong

**Affiliations:** 1 Guangdong Provincial Key Laboratory of Veterinary Pharmaceutics Development and Safety Evaluation, South China Agricultural University, Guangzhou, China; 2 National Risk Assessment Laboratory for Antimicrobial Resistance of Animal Original Bacteria, South China Agricultural University, Guangzhou, China; 3 National Laboratory of Safety Evaluation (Environmental Assessment) of Veterinary Drugs, South China Agricultural University, Guangzhou, China; 4 National Reference Laboratory of Veterinary Drug Residues, South China Agricultural University, Guangzhou, China; Kansas State University, Manhattan, Kansas, USA

**Keywords:** whole-genome sequence analysis, genetic diversity, toxins, antimicrobial resistance, public health

## Abstract

*Bacillus cereus*, a global threat, is one of the major causes of toxin-induced foodborne diseases. However, a comprehensive assessment of the prevalence and characteristics of *B. cereus* worldwide is still lacking. Here, we applied whole-genome sequence analysis to 191 *B. cereus* collected in Africa, America, Asia, Europe, and Oceania from the 1900s to 2022, finding that CC142 dominated the global *B. cereus* clonal complex. The results provided direct evidence that *B. cereus* could spread through the food chain and intercontinentally. *B. cereus* from different generations worldwide showed coherence in the antibiotic-resistant gene and virulence and biofilm gene profiles, although with high genomic heterogeneity. The *BCI-BCII-vanZF-fosB* profiles and virulence and biofilm genes were detected at high rates, and we emphasized that *B. cereus* would pose a serious challenge to global public health and clinical treatment.

**IMPORTANCE:**

This study first emphasized the prevalence, genetic characteristics, and pathogenicity of *Bacillus cereus* worldwide from the 1900s to 2022 using whole-genome sequence analysis. The CC142 dominated the global *Bacillus cereus* clonal complex. Moreover, we revealed a close evolutionary relationship between the isolates from different sources. *B. cereus* isolates from different generations worldwide showed coherence in potential pathogenicity, although with high genomic heterogeneity. The *BCI-BCII-vanZF-fosB* profiles and virulence and biofilm genes were detected at high rates, and we emphasized that *B. cereus* would pose a serious challenge to global public health and clinical treatment.

## INTRODUCTION

*Bacillus cereus sensu stricto* (herein referred to as *Bacillus cereus*) is a ubiquitous, highly drug-resistant bacterium known for causing food poisoning. It poses a significant problem for food safety and public health. *B. cereus* is a Gram-positive bacteria capable of producing spores, enabling it to survive in harsh environments (1). This is why preventing *B. cereus* contamination in food products is almost impossible (2, 3). An estimated 1.4%–12.0% of global foodborne outbreaks can be attributed to *B. cereus* (4). *B. cereus* is the second and third most common causative agent of foodborne diseases in France and Europe, respectively (5, 6). Similarly, a 10-year survey reported that *B. cereus* was the third leading foodborne pathogen in China (7). The primary food poisoning caused by *B. cereus* includes two main types: diarrhea and emetic syndrome (8). The diarrheal type results from enterotoxins produced by *B. cereus* growing in the gut (a toxico-infection), while the emetic type is caused by cereulide toxin produced in contaminated food before it is consumed (an intoxication). In addition, *B. cereus* can also cause severe diseases, such as endocarditis, endophthalmitis, respiratory tract infection, and sepsis, leading to multiple organ failure and even death (9, 10). Therefore, it is essential to understand and evaluate the safety of *B. cereus* worldwide.

*B. cereus* in the *B. cereus* group is a source of foodborne disease outbreaks. The *B. cereus* group consists of several genetic proximity species, and species differentiation within the group is complex (11). However, the *B. cereus* group members could not be distinguished only by phenotypic differences, distinct virulence trait, and extrachromosomal elements (12). The whole-genome sequencing (WGS) classification methods, such as *rpoB* housekeeping gene, *panC* phylogenetic group assignment, average nucleotide identity (ANI) framework classification, and Genome Classification Database (GTDB) species assigned, are highly efficient and accurate (13–15). Therefore, the above classification methods were integrated to identify *B. cereus* to avoid taxonomic ambiguity and maximize acceptability in this study.

The pathogenicity of *B. cereus* is closely related to the diversity of virulence genes it carries (16). First, the cereulide toxin associated with the *ces* gene cluster is worth our attention. The cereulide toxin is highly heat and acid resistant, resulting in its stable residue in food and processing lines, despite killing the bacteria (3). In addition, the heat-labile enterotoxins responsible for *B. cereus* diarrhea syndrome: nonhemolytic enterotoxin (*Nhe*), hemolysin BL (*Hbl*), cytotoxin K (*CytK*), enterotoxin FM (entFM), and potential enterotoxin hemolysin II (*HlyII*) (17). Furthermore, *B. cereus* can produce biofilm, which is also an essential factor in bacterial virulence (18). Research has shown that bacteria can exchange virulence genes with the help of biofilms and develop new pathogenic properties (19). In prolonged antibiotic use and abuse, highly adaptable *B. cereus* isolates most likely develop drug resistance rapidly. Studies have reported the widespread presence of multidrug-resistant *B. cereus* strains, and their antibiotic-resistant genes (ARGs) can be transferred horizontally by plasmids and transposons (20–22). Under the food poisoning and severe infections caused by highly adaptive *B. cereus*, if antibiotic treatment fails, it will greatly increase the risk to public safety. Therefore, it is urgent to continuously monitor the ARG and virulence and biofilm genes of *B. cereus*.

Recently, *B. cereus* has been widely studied worldwide (21, 23–25). However, there has been no comprehensive assessment of the prevalence and characteristics of *B. cereus* worldwide. This study provides an assessment of the genetic characteristics and pathogenicity of *B. cereus* based on WGS.

## MATERIALS AND METHODS

### Sample collection

We conducted a search using “*Bacillus cereus*” as the keyword in the National Center for Biotechnology Information (NCBI) and downloaded all the available genome assemblies pertaining to *B. cereus* (*n* = 1246) from the “Assembly/Genome NCBI Datasets” section (April 2022). Additional genomes (*n* = 37) were provided from our previous study (21).

### Whole-genome analysis

Raw reads (scores <20 or length <20 bp) were discarded using Sickle (v.1.3.3). Each genome was assembled *de novo* with the default k-mer size using the CLC Genomics Workbench (v.10.0.1; CLC Bio, Aarhus, Denmark). The quality of each genome was assessed using QUAST (v.4.5), and the contamination and completeness of the genome were assessed using CheckM (v.1.1.3). The genome (completeness ≥97.5%, contamination ≤2.5%) with less than 1,000 contigs and N50 greater than 20 kbp was retained for downstream analysis. The FastANI (v.1.0) and GTDB-Tk (v.1.3.0) were used to identify *B. cereus* from the *B. cereus* group (26, 27). BTyper (v.3.1.1) was used to classify the *panC* group, *rpoB* allelic typing (13), the multilocus sequence typing (MLST) profiles, and the clonal complex (CC) associated with the PubMLST latest *B. cereus* database (https://pubmlst.org). Detailed information is provided in Tables S1 and S2.

We annotated the draft bacterial genomes using Prokka (v.1.14.6) (28). The ARG identified against the CARD, ARG, and resfinder database (data sets updated on 10 October 2022) using ABRicate (v.1.0.1) (https://github.com/tseemann/abricate) with default setting (identity ≥80% and query coverage ≥80%). The virulence gene database of the *B. cereus* group in BTyper (v.3.1.1) was used to detect the virulence genes (parameters: default).

### Phylogenetic analysis

The single-nucleotide polymorphisms (SNPs) and the cleaning of the entire genome alignment were generated by Snippy (v.4.3.6) (https://github.com/tseemann/snippy). After detecting recombination (Gubbins, v.2.4.1) (29), the maximum-likelihood phylogenetic tree was constructed by RAxML (v.2.0.3) using the model of GTRGAMMA with 1,000 bootstraps (30). HierBAPs (v.6.0) was used to identify population structures with Bayesian models (31). The phylogenetic tree was annotated using iTol (v.6.8). The minimum spanning tree of MLST was generated in PHYLOViZ (v.2.0).

### Statistical analyses

The map was created using the generator at https://pixelmap.amcharts.com/. The heatmap and rainfall chart were generated using the “pheatmap” and “gghalves” packages, respectively. We identified the pairwise co-occurrence and mutual exclusivity of ARG, virulence, biofilm, and insertion sequence (IS) genes in the genomes using the R package Discover (v.0.9.4) (32). The Kruskal-Wallis tests were employed to compare the data differences. A *P* value of <0.05 was considered statistically significant. Data visualization was performed using RStudio (v.4.2.0).

## RESULTS

### Prevalence and taxonomic classification

**Fig 1:**
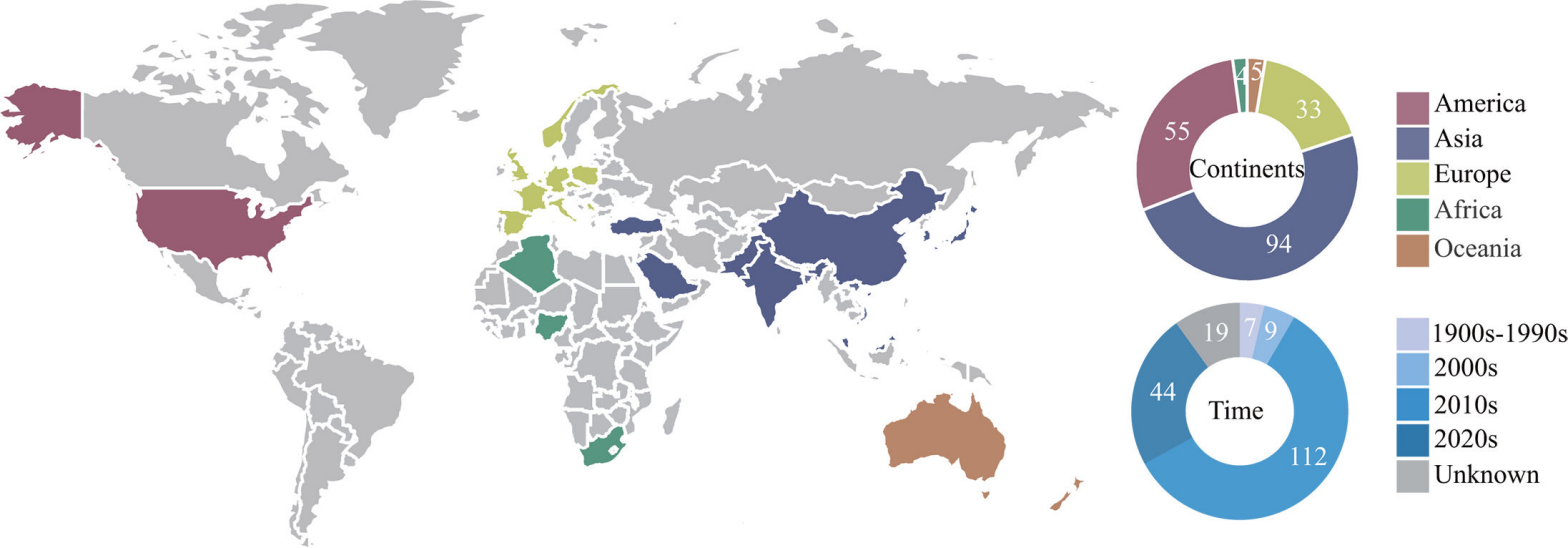
Distribution map of *B. cereus* genomes overall. The pie chart shows the distribution of *B. cereus* across continents and time.

Only 217 of the 1246 genomes deposited in the NCBI databases were identified as *B. cereus sensu stricto* using the ANI alignment scheme and GTDB-Tk until April 2022. After quality control (completeness ≥97.5%, contamination ≤2.5%), 191 isolates (37 from our laboratory and 154 from the NCBI databases) were retained for downstream analysis, which was annotated as *B. cereus* in both ANI alignment and GTDB classification (Tables S1 and S2). Moreover, *panC* gene sequences showed that all 191 isolates belong to phylogenetic group IV (Table S3). Isolates were collected from Africa (*n* = 4, 2.1%), America (*n* = 55, 28.8%), Asia (*n* = 97, 50.8%), Europe (*n* = 33, 17.3%), and Oceania (*n* = 2, 1.0%). All isolates were chronologically divided into four periods: 1900s–1990s (*n* = 7, 3.7%), 2000s (*n* = 9, 4.7%), 2010s (*n* = 112, 58.6%), and 2020s (*n* = 44, 23.0%) (Fig. 1).

### Multilocus sequence type analysis

**Fig 2:**
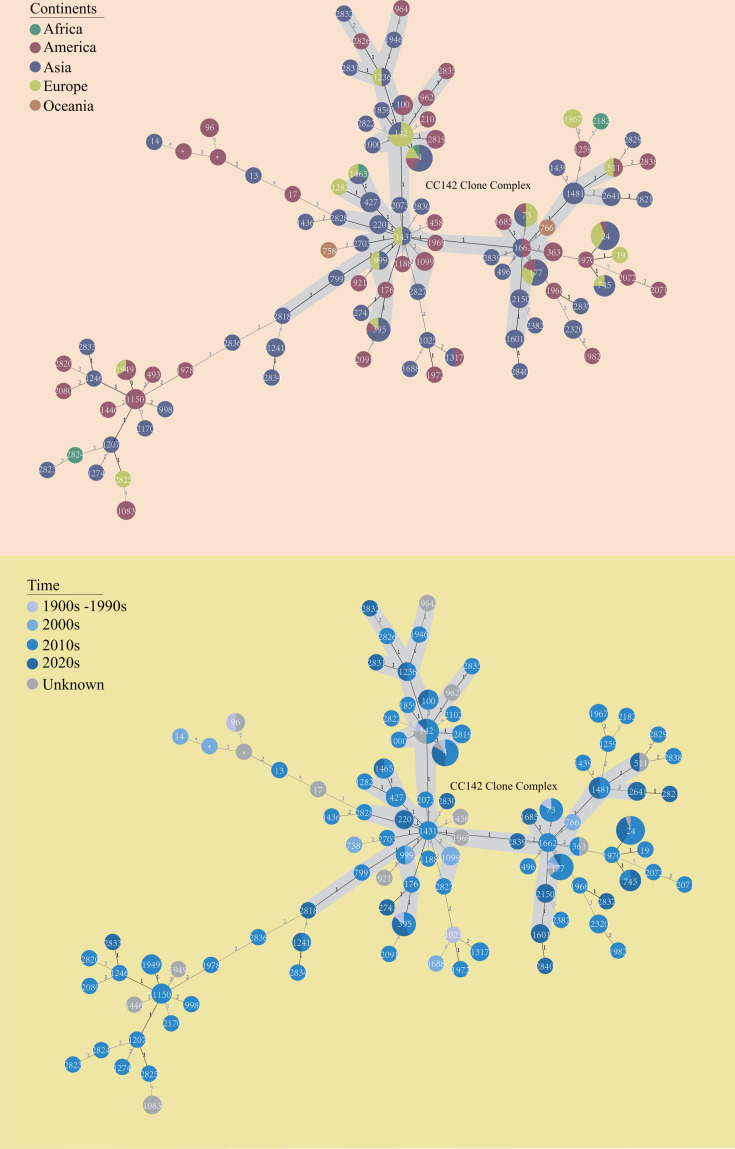
Minimum spanning tree and genetic diversity of *B. cereus*. The colors of the circles correspond to the origin of different continents and times. Each circle represents isolates with an allelic profile based on the MLST. Clusters of closely related isolates are shaded in gray. The size of the circle is proportional to the number of isolates it represents. Branch lengths between circles are proportional to the number of alleles that differ between the two linked circles.

MLST revealed the genetic diversity of *B. cereus* based on internal sequence analyses of seven housekeeping genes (Fig. 2). Multilocus sequence typing assigned the 191 isolates to 100 distinct sequence types (STs) (Table S3). The ST24 (8.9%, *n* = 17, the 2010s–2020s), ST4 (6.3%, *n* = 12, the 1900s–1990s and the 2010s–2020s), and ST177 (5.8%, *n* = 11, the 1900s–2020s) were the dominant STs, while others were less than 5%. CC CC142 was the only one identified and contained 87 strains. Additionally, over half of the STs (54.5%, *n* = 104) were not assigned in PubMLST. We identified 16 STs from 86 (45.0%) isolates, which were present on at least two continents, including ST24 (*n* = 17), ST4 (*n* = 12), ST177 (*n* = 11), ST142 (*n* = 8), and ST395 (*n* = 7). Furthermore, ST1150 (5.5%, *n* = 3, the 2010s) and ST24 (10.3%, *n* = 10, the 2010s–2020s) were the prevailing STs in America and Asia, respectively. In Europe, ST142 (18.2%, *n* = 6, the 1900s–1990s and the 2010s) and ST24 (18.2%, *n* = 6, the 2010s) were the prevalent STs.

### Phylogenetic analysis of 191 *B. cereus* genomes

**Fig 3:**
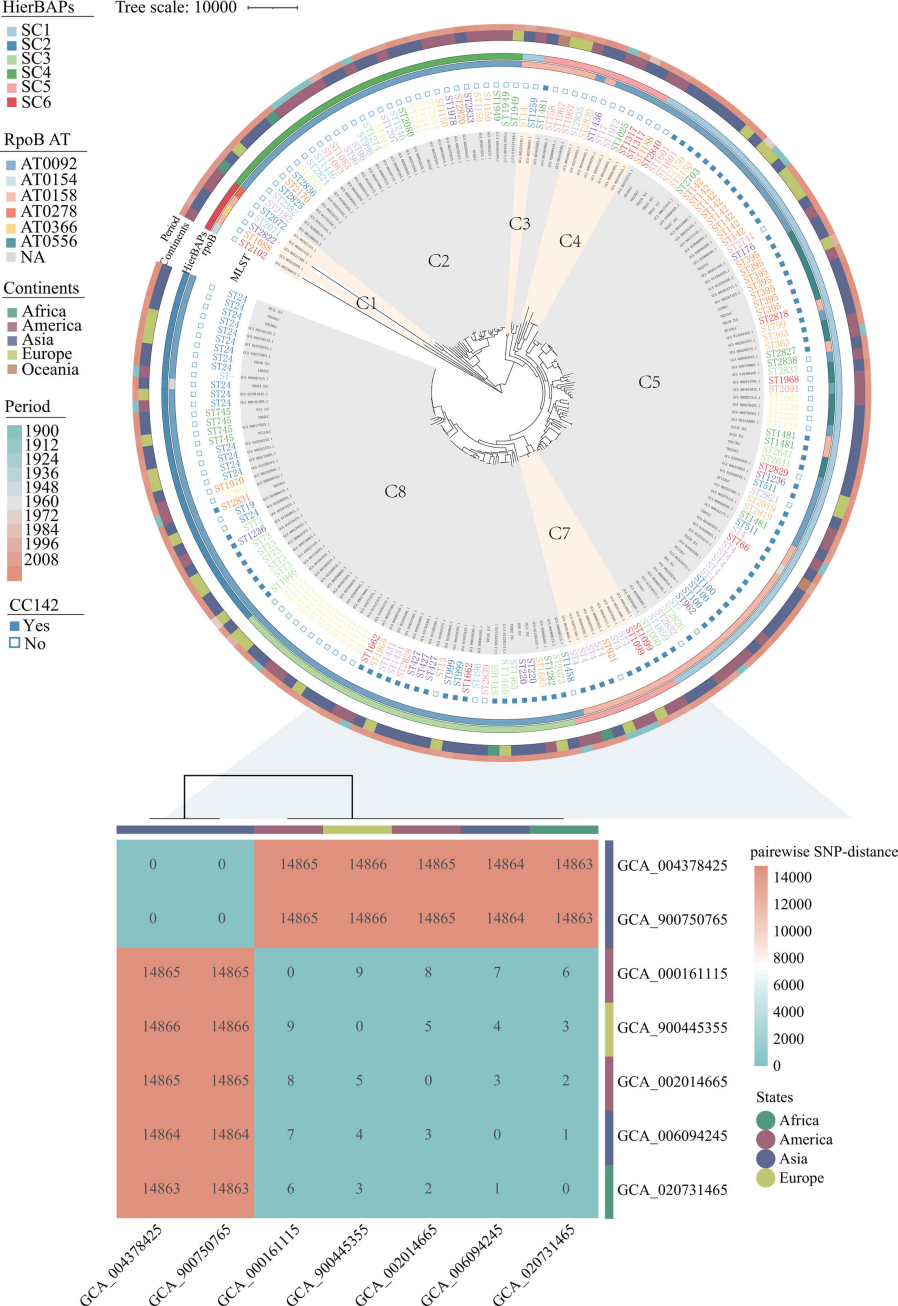
A maximum likelihood phylogeny constructed using core-genome SNPs of 191 *B. cereus* isolates worldwide. The ring from inner to outer indicates the source, *rpoB* allelic typing, the Bayesian hierarchical clustering analysis lineages, continents of isolations, and the year of collection. The heatmap shows the pairwise SNP distance of seven *B. cereus* isolates with close genetic relationships.

Among the 191 bacterial genomes analyzed in our study, a total of 40,512 genes were identified, of which 2,868 genes constituted the core genome. A maximum likelihood phylogeny with eight clades was constructed using core-genome SNPs to investigate further the genetic characteristics of *B. cereus* of isolates collected worldwide (Fig. 3). *B. cereus* genome was highly heterogeneous, with pairwise SNP distances ranging from 0 to 58,159 (Table S4). In addition, the eight clades (C1–C8) comprised 2.6%, 15.7%, 1.0%, 2.1%, 4.2%, 34.6%, 4.7%, and 35.1%, respectively. Phylogenetic and genetic analyses showed substantial diversity within the subject isolates and identified six sequenced clusters (SCs) using Bayesian model-based population structure analysis (SC1–SC6), with population sizes of 64, 34, 33, 30, 22, and 5, respectively. All SCs comprised isolates from different periods. Detailed analysis showed that *B. cereus* isolates from Asia (dominated SCs: SC1 and SC3, 40.2% and 20.6%) and America (dominated SCs: SC1 and SC3, 32.7% and 25.5%) could be observed in every SCs but exhibited a high degree of genetic diversity, and isolates from Europe were detected in all SCs except SC6 (dominated SCs: SC1 and SC2, 27.3% and 33.3%). Notably, the heatmap of pairwise SNP-distance revealed that seven *B. cereus* isolates from separate continents (Africa, America, Asia, and Europe) exhibited close genetic relationships. For example, GCA_900750765 (Asia, Israel, roots of *Coriandrum sativum*) and GCA_004378425 (Asia, Israel, roots of *Coriandrum sativum*) had no pairwise SNP distance in C6 (SC1, AT0566 and ST2320). Isolates of GCA_006094295 (Asia, China) and GCA_020731465 (Africa, Pretoria) exhibited only one pairwise SNP distance in C7 (SC5, AT0158 and ST4). Similarly, isolates of GCA_002014665 (America, USA) and GCA_020731465 (Africa, Pretoria) exhibited two pairwise SNP distances in C7 (SC5, AT0158 and ST4).

### Temporal and spatial variations in ARG, virulence gene, and biofilm gene profiles

**Fig 4:**
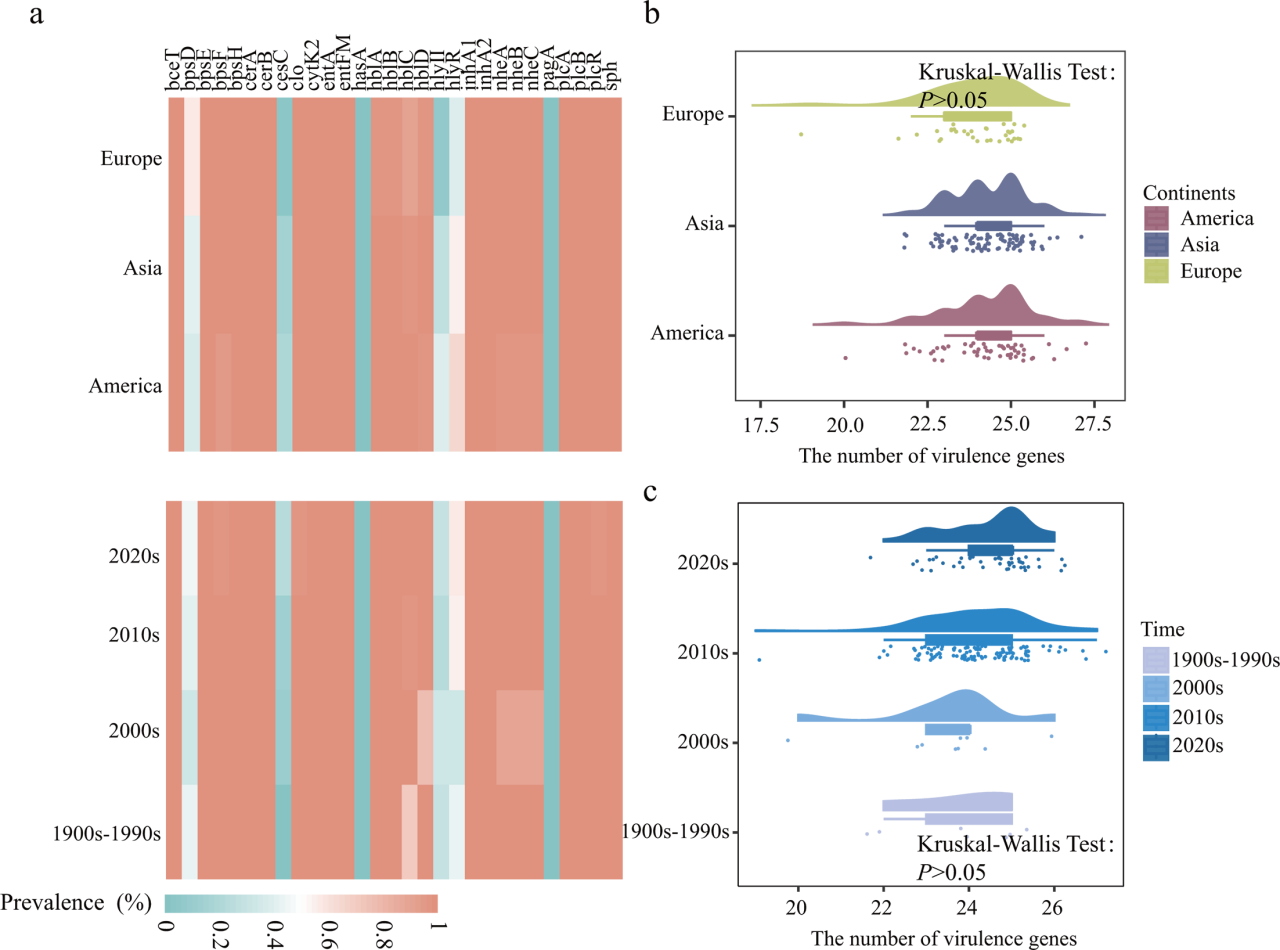
Comparison of virulence genes in *B. cereus* from different continents and generations. (**a**) The prevalence of virulence genes in *B. cereus* from different continents and generations. (**b**) The number of virulence genes in *B. cereus* from different continents and (**c**) generations. Kruskal-Wallis tests were used to determine significant differences. A *P* value of <0.05 was considered statistically significant.

We identified 29 virulence genes using BTyper, and the average *B. cereus* contains 24 virulence genes (Fig. 4a; Table S5). Notably, the enterotoxin genes (*entFM*, *entA*, *nheA*, *nheB*, *nheC*, *hblA*, *hblB*, *hblC*, and *hblD*), cytotoxin genes (*cytK-2*), and diarrhea toxin genes (*bceT*), which were closely associated with *B. cereus* food poisoning, were all present at high prevalence in America, Asia, and Europe, ranging from 90.9% to 100.0%. The prevalence of *cesC* (enterotoxin gene) gradually increased over the 1900s–2020s, from 0% to 22.7%. Similarly, there was no significant difference in the number of virulence genes among America, Asia, and Europe from the 1900s to 2020s (*P* > 0.05, Fig. 4b and c).

We identified 25 genes involved in forming biofilms from the annotation in *B. cereus* draft genomes (Table S6). We identified *clpY*, *codY*, *sigB*, *sinR*, *sipW*, *tasA*, *xerC*, and the *pur* gene clusters (*purA*, *purB*, *purC*, etc.) with high prevalence (96.7%–100.0%) in *B. cereus* from America, Asia, and Europe (Fig. S1a). Particularly, five genes (*epsD*, *epsG*, *epsK*, *epsM*, and *epsO*) appeared in the 2010s, with a prevalence from 0.9% to 6.8% between the 2010s and 2020s. No systematic differences in biofilm gene numbers were observed among America, Asia, and Europe from the 1900s to 2020s (*P* > 0.05, Fig. S1b and c).

In total, seven ARGs assigned into four types (β-lactam, fosfomycin, tetracycline, and glycopeptide) were identified in all subject isolates, and the ARG profile of *BCI-BCII-vanZF-fosB* (86.4%, *n* = 165) was prevalent in America, Asia, and Europe between the 1900s and 2020s (Fig. S2a; Table S7). There was no significant difference in the number of ARGs among America, Asia, and Europe from the 1900s to 2020s (*P* > 0.05, Fig. S2b and c). Generally, the ARG, virulence gene, and biofilm gene profiles were similar among isolates from different continents in the 1900s–2020s.

### Co-occurrence of ARG, virulence genes, biofilm genes, and ISs

**Fig 5:**
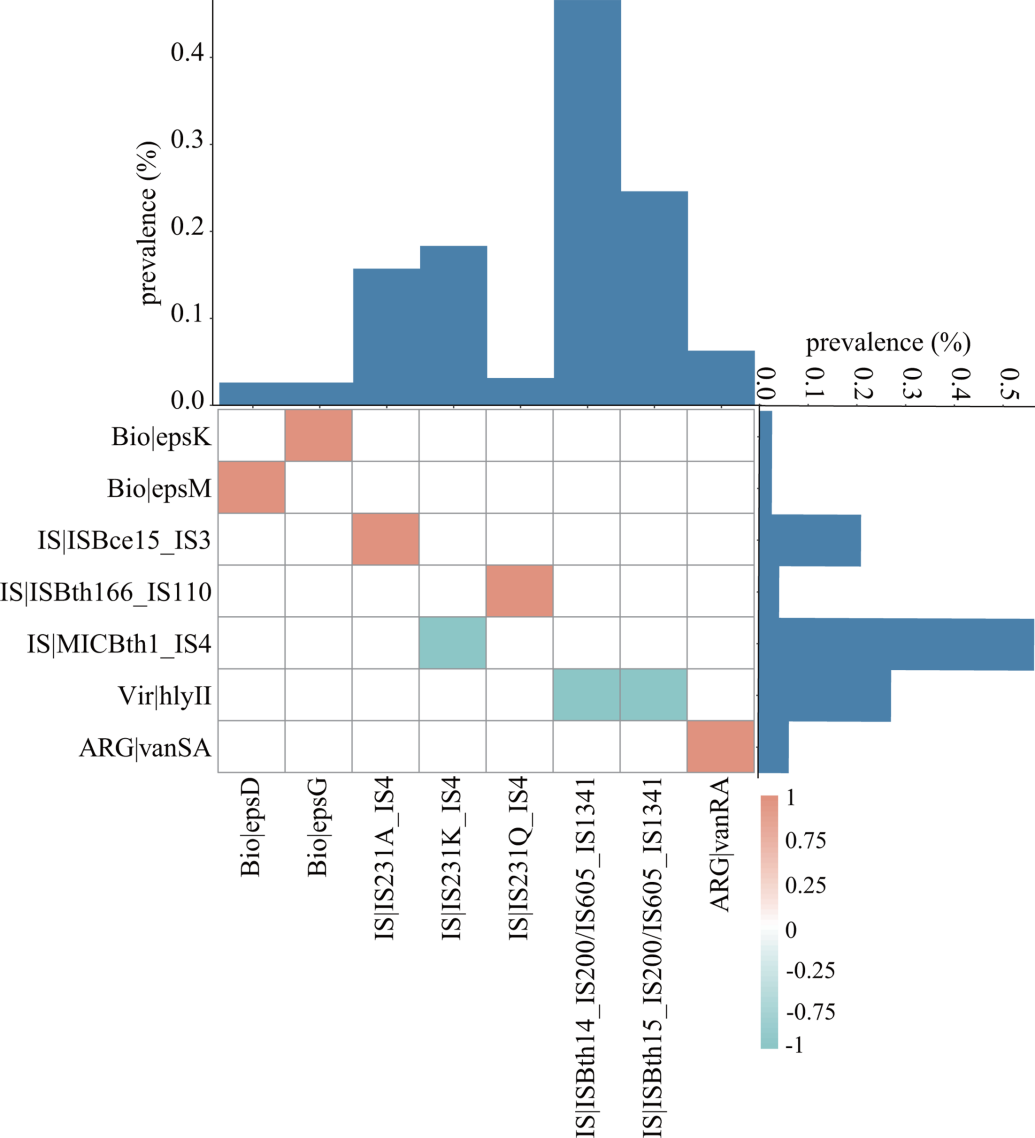
The co-occurrence and mutual exclusivity of ARG, virulence and biofilm genes, and ISs. We defined the false discovery rate threshold as 0.05 in this algorithm, and significant pairwise values from 1 (orange) to −1 (green) (i.e., co-occurrence and mutual exclusivity) are displayed. The bar plot demonstrates the prevalence of ARG and virulence and biofilm genes and ISs in 191 *B. cereus* isolates.

The co-occurrence and mutual exclusivity were determined to illustrate the complex relationships among the individual ARG, virulence genes, biofilm genes, and ISs (Fig. 5) (ISs information provided in Table S8). The ARG of *VanRA* (12 of 191) co-existed with *VanSA* (12 of 191). We found that the biofilm genes of *epsk* co-existed with *epsG*, as well as the *epsM* and *epsD* genes. Interestingly, the virulence gene of *hlyII* was mutually exclusivity with IS*Bth14*_IS*200*/IS*605*_IS*1341* and IS*Bth15*_IS*200*/IS*605*_IS*1341*. However, no co-occurrence and mutual exclusivity factors were identified when the analysis was restricted to different continents or chronological groups. Significant correlations between ARG subtypes and ISs in *B. cereus* were not observed; therefore, the assessment of potential horizontal transfer risk remains limited.

## DISCUSSION

This study presents a surveillance report about the genetic characteristics in *B. cereus* from 1900 to 2022 worldwide. The high genome similarity within the *B. cereus* group makes taxonomic classification difficult to discern (33). We used the ANI and GTDB, and only 191 *B. cereus sensu stricto* spp. were identified. Our research showed that the prevalence of *B. cereus* was highest in Asia (50.1%), followed by the Americas (28.8%), Europe (17.3%), and Africa (2.1%), and the lowest in Oceania (1.0%). A recent survey on the prevalence of *B. cereus* in food showed that Asia was second to the Americas, with the lowest prevalence in Australia (Oceania) (25). In this study, the source of isolates not being strictly restricted to food may be the reason for the difference between the previous results. In addition, the extremely high prevalence of *B. cereus* (58.6%) during the 2010s may suggest an outbreak event during this period. National surveillance data showed an overall increase trend caused by bacterial toxins, and numerous countries, for example, Austria, China, and France, reported *B. cereus* outbreaks in food throughout the 2010s (34–36).

MLST-based genotyping is more conducive to revealing the molecular characteristics of *B. cereus* and facilitating the traceability of pathogens (37). *B. cereus* has high genetic heterogeneity (38), and we identified 100 STs from 191 *B. cereus* spp. However, CC142 was the only major prevalent clonal complex identified in subject continents from the 1900s to the 2020s. CC142 has often been associated with clinical cases in existing studies (39, 40). This result indicated that CC142 was stable and widely existed in different continents, which would pose a serious threat to the global environment and public health. Moreover, we identified two *B. cereus* spp. from Europe and Asia that shared nearly identical core genomes with one from Africa. In addition, we found that two *B. cereus* spp. that shared identical core genomes in Africa were both from vegetables. These *B. cereus* spp. from different spatiotemporal origins indicate that a close evolutionary relationship exists between the isolates from different sources.

Antimicrobial-resistant pathogens can be transmitted along the food chain and thus increase the invasive infection and the risk of death for the consumer. In recent years, *B. cereus* has developed varying degrees of resistance to numerous antibiotics, posing a severe barrier to therapeutic treatment (41). The *BCI-BCII-vanZF-fosB* profile was present in 86.4% of *B. cereus* from America, Asia, and Europe in the 1900s and 2020s. In the previous study, we showed that *B. cereus* spp. carrying the *BCI-BCII-vanZF-fosB* profile were all resistant to penicillin, amoxicillin, cefoxitin, ceftiofur, sulfamethoxazole-trimethoprim, and tiamulin (21). *B. cereus* was typically resistant to β-lactams, and in recent years, *B. cereus* has also been reported to be a highly multidrug resistant (42–45). Notably, the number of ARGs in *B. cereus* was highly stable in America, Asia, and Europe from the 1900s to the 2020s, and no evidence of horizontal gene transfer was found in our study. Even though *B. cereus* spp. have been shown to exchange plasmids with *B. thuringiensis* (46), few studies have provided direct evidence for horizontal transfer of ARG in *B. cereus*.

*B. cereus* can cause food poisoning and other infections, such as emetic and diarrheal syndromes and bacteremia (47, 48). The pathogenesis of B. cereus to produce enterotoxin and hemolysin, such as Hbl, Nhe, and CytK, was observed with high detection rates (90.9%–100%) in America, Asia, and Europe from the 1900s to 2020s. A previous study reported that *B. cereus* CC142 harbored *nheABC*, *hblCDA*, and *cytK2* and exhibited cytotoxicity (49). In this study, the positive rate of *cesC* toxin has gradually increased with time. The localized increase of this gene over time could represent vertical expansion of the lineage containing *cesC*. *cesC* participates in the efflux of cereulide toxin and endows it with self-resistance, and the attachment and accumulation of this toxin will further increase the risk of food safety (50, 51). Biofilms can promote the colonization and growth of bacteria in the host, thereby increasing their pathogenicity (52). The biofilm genes of *B. cereus* in different times and regions had a high detection rate, consistent with previous studies (23), and the virulence genes had co-occurrence. In general, the high prevalence of *B. cereus* virulence and biofilm genes makes it more susceptible to cause foodborne diseases and less susceptible to immune attack and antibiotics, posing a significant challenge to public health and therapy in the future.

We should acknowledge several limitations of the study. First, our analysis was not as comprehensive as expected because not all *B. cereus* genomes in the NCBI database were included in this study. Second, we could not further analyze the evolutionary relationships of these *B. cereus* due to the incomplete metadata. Third, this study includes 37 genomes from our laboratory, while the remaining strains are from the NCBI public database. We recognize that this combination might affect the generality and representativeness of the results. However, our preliminary analysis indicates that these laboratory-preserved genomes do not show significant genomic differences compared to the other strains downloaded from NCBI. Nonetheless, we recommend considering this factor when interpreting the results. Future studies should incorporate more strains from different geographical locations and environments to further validate our conclusions.

### Conclusion

The CC142 dominated the global *B. cereus* clonal complex. Despite the observed high genomic heterogeneity, the ARG, virulence gene, and biofilm gene profiles of *B. cereus* isolates across different generations exhibit a striking degree of similarity globally. The high detection rates of *BCI-BCII-vanZF-fosB* profile and virulence and biofilm genes of *B. cereus* in different years worldwide would pose a serious challenge to global public health and clinical treatment. This research enhanced our understanding of worldwide contamination and genomic characteristics of *B. cereus* isolates and provided a theoretical basis for global contamination risk assessment and safety management.

## Data Availability

Data will be made available on request.

## SUPPLEMENTAL MATERIAL

Supplemental figures msphere.00702-24-s0001.docx Figures S1 and S2.

Supplemental tables msphere.00702-24-s0002.xlsx Tables S1 to S8.

ASM does not own the copyrights to Supplemental Material that may be linked to, or accessed through, an article. The authors have granted ASM a non-exclusive, world-wide license to publish the Supplemental Material files. Please contact the corresponding author directly for reuse.
