# Supplementary material for: First insights into the prevalence, genetic characteristics, and pathogenicity of Bacillus cereus from generations worldwide
Source: mSphere. 2024 Oct 23;9(11):e00702-24. doi: 10.1128/msphere.00702-24 (PMC11580406; doi:10.1128/msphere.00702-24)
Supplement: Supplemental figures — Figures S1 and S2. [file msphere.00702-24-s0001.docx]

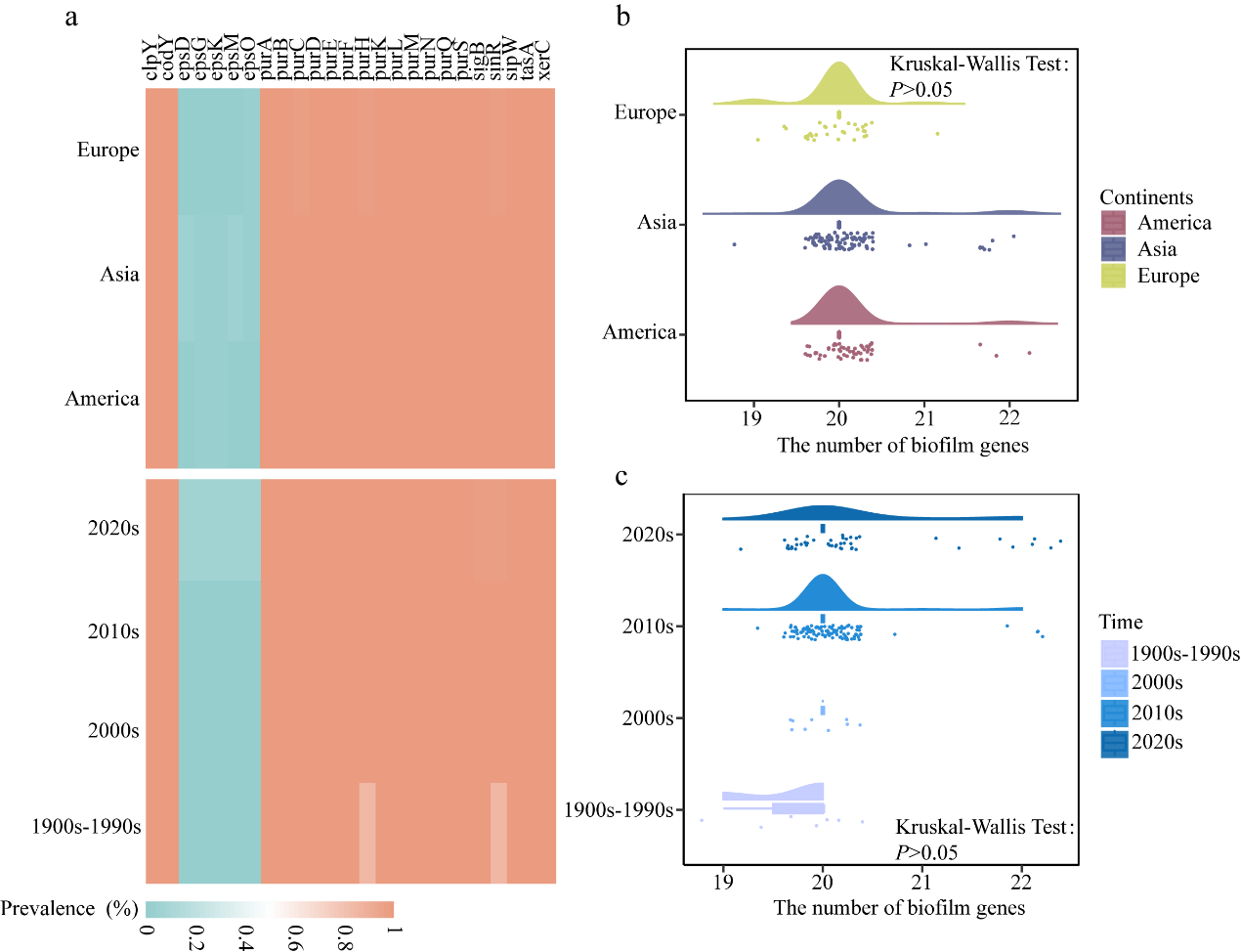


Supplemental Figure 1: Comparison of biofilm genes in B. cereus from different continents and generations. (a) The prevalence of biofilm genes in B. cereus from different continents and generations. (b) The number of biofilm genes in B. cereus from different continents and (c) generations. Kruskal-Wallis tests were used to determine significant differences. P<0.05 was considered statistically significant.


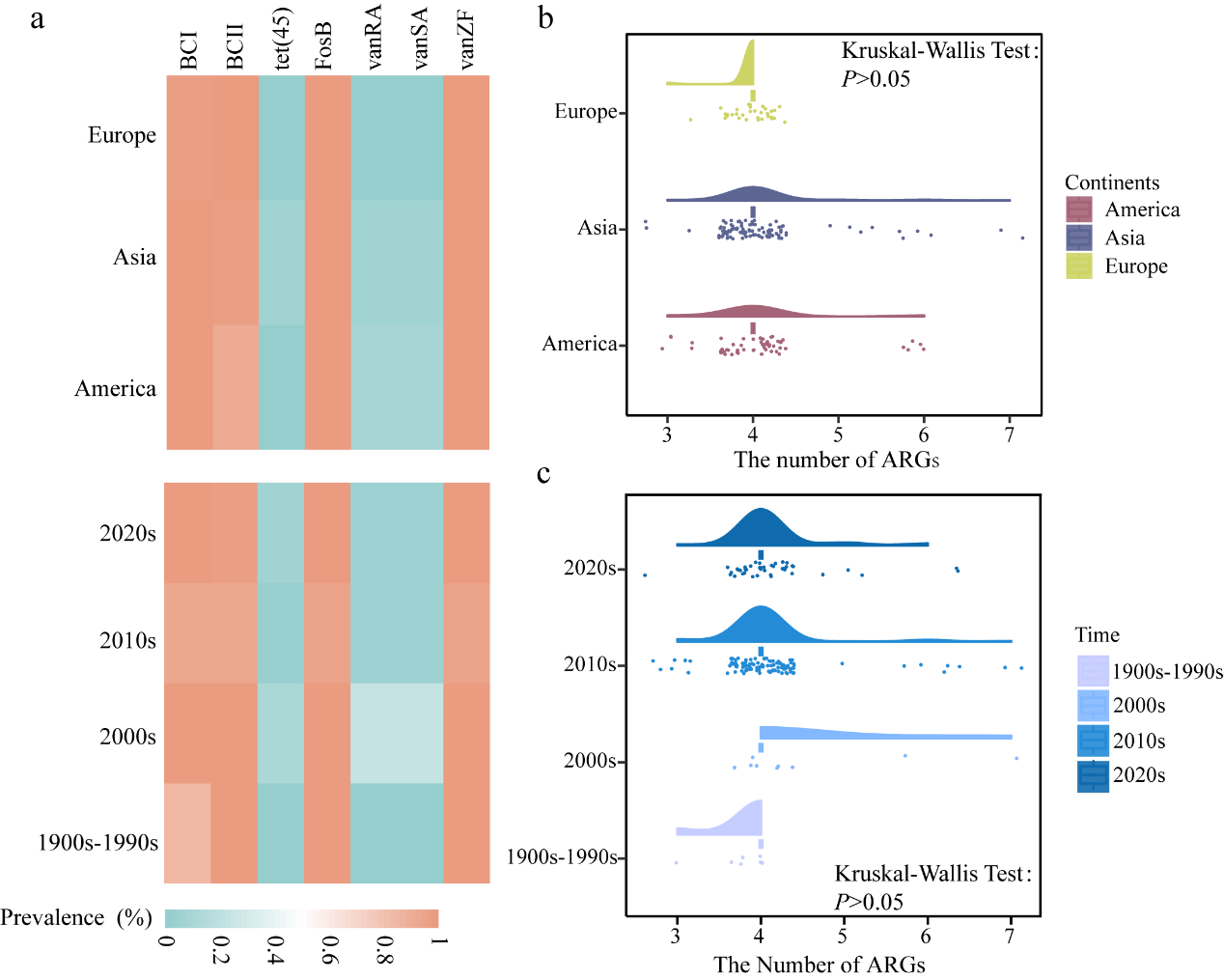


Supplemental Figure 2: Comparison of ARGs in B. cereus from different continents and generations. (a) The prevalence of ARGs in B. cereus from different continents and generations. (b) The number of ARGs in B. cereus from different continents and (c) generations. Kruskal-Wallis tests were used to determine significant differences. P<0.05 was considered statistically significant.
